# Predicting overall survival in synchronous metastatic nasopharyngeal carcinoma using a stacking ensemble machine learning model: a multicenter retrospective study

**DOI:** 10.3389/fonc.2026.1866690

**Published:** 2026-07-01

**Authors:** Canwen Che, Shanyue Lin, Jie Ma, Haibo Liu, Wentao Liu, Zhanhong Tan, Xin Chen, Xiaoyi Zeng, Qiwen Duan, Guanxun Cheng

**Affiliations:** 1 Shantou University Medical College, Shantou, China; 2 Department of Radiation Oncology, The People’s Hospital of Baoan Shenzhen, Shenzhen, China; 3 Department of Radiology, Xiangtan Central Hospital, Xiangtan, Hunan, China; 4 Medical Imaging Department, Guangxi Medical University Cancer Hospital, Nanning, Guangxi, China; 5 Department of Radiation Oncology, Wuzhou Red Cross Hospital, Wuzhou, Guangxi, China; 6 Department of Radiology, Peking University Shenzhen Hospital, Shenzhen, China

**Keywords:** machine learning, nasopharyngeal carcinoma, SHAP, stacking ensemble, synchronous metastasis

## Abstract

**Background:**

Patients with synchronous metastatic nasopharyngeal carcinoma (smNPC) show marked survival heterogeneity, which complicates individualized prognostic assessment and treatment planning. We developed and validated a stacking ensemble machine learning model for predicting 3-year overall survival (OS) in this population.

**Methods:**

This multicenter retrospective study included 413 patients from three institutions, randomly assigned to a training set (n = 289) and an internal held-out validation set (n = 124). Candidate predictors included demographic, histopathologic, serological, and treatment-related variables. The 3-year OS label was assigned only when death occurred within 3 years or when follow-up confirmed survival at 3 years. Six base learners were trained: gradient boosting decision tree (GBDT), adaptive boosting (AdaBoost), extreme gradient boosting (XGBoost), Light Gradient Boosting Machine (LightGBM), categorical boosting (CatBoost), and random forest (RF). Hard voting, soft voting, and stacking ensembles were constructed and compared. Discrimination, calibration, decision curve analysis, leave-one-center-out (LOCO) validation, overfitting diagnostics, and Shapley Additive Explanations (SHAP) were assessed.

**Results:**

The stacking ensemble achieved the highest internal held-out validation AUC of 0.8358 for 3-year OS prediction. The 3-year death event rate was 60.0% overall, 59.9% in the training set, and 60.5% in the internal held-out validation set. After multiplicity adjustment, stacking showed significantly higher AUC than GBDT, AdaBoost, and hard voting, whereas differences versus other comparators were not significant. Overfitting diagnostics showed a training AUC of 0.9150, an optimism gap of 0.0792, and an optimism-corrected AUC of 0.8415. SHAP identified immunotherapy, first-line regimen, number of metastatic lesions, and number of metastatic organs as influential predictors.

**Conclusion:**

The stacking ensemble model provided an interpretable approach for individualized 3-year OS prediction in smNPC and may support multidisciplinary prognostic assessment, although prospective validation remains needed.

## Introduction

Nasopharyngeal carcinoma is endemic in southern China and Southeast Asia and shows a greater propensity for distant dissemination than most other head and neck malignancies (1). Synchronous metastatic nasopharyngeal carcinoma (smNPC), defined as distant metastasis at the time of initial diagnosis, accounts for approximately 4% to 10% of newly diagnosed cases (2, 3). The bone, lung, and liver are the most common metastatic sites. Despite advances in systemic therapy and radiotherapy, the prognosis of this population remains poor, with median overall survival generally ranging from 12 to 30 months (4, 5).

Patients with smNPC show substantial survival heterogeneity. Outcomes are influenced by demographic characteristics, tumor burden, metastatic distribution, blood-based biomarkers, and multimodality treatment strategies (6). Recent studies have shown that first-line immunochemotherapy (7), radiotherapy to metastatic lesions (8, 9), and locoregional radiotherapy to the primary site (10) may improve outcomes in selected patients. In addition, 3-year OS has emerged as a clinically meaningful endpoint for prognostic assessment in this setting (11). However, current risk stratification remains imperfect, particularly when multiple interacting predictors must be considered simultaneously.

Conventional statistical models and single machine learning algorithms may not fully capture nonlinear associations or higher-order interactions within heterogeneous clinical datasets. Ensemble learning offers a practical way to address this limitation by integrating complementary predictive patterns from different base learners. Among ensemble methods, stacking uses a meta-learner to combine the outputs of several base models and can therefore improve robustness and discrimination when the base learners provide nonredundant information (12–14).

The aim of this study was to develop and validate a stacking ensemble machine learning model for predicting 3-year OS in patients with smNPC using data from a multicenter retrospective cohort. We also compared stacking with individual machine learning models and alternative ensemble strategies and used SHAP to improve model interpretability.

## Materials and methods

### Study design and population

This multicenter retrospective study consecutively included patients with smNPC who received multimodality treatment between January 2010 and January 2023 at three participating institutions. Eligible patients met the following criteria: (1) histopathologically confirmed nasopharyngeal carcinoma; and (2) distant metastasis at initial diagnosis confirmed either by biopsy of metastatic lesions or by imaging examinations, including computed tomography, chest radiography, abdominal ultrasonography, magnetic resonance imaging, bone scintigraphy, or positron emission tomography and computed tomography. Patients were excluded if they met any of the following criteria: (1) insufficient follow-up to determine 3-year OS status among patients alive at the last contact; (2) another malignant tumor; (3) uncontrolled cardiac, pulmonary, renal, or hepatic disease; (4) refusal of all antitumor treatment; or (5) receipt of fewer than four cycles of chemotherapy. Accordingly, patients were labeled as 3-year death events if death from any cause occurred within 3 years after diagnosis, and as 3-year survivors only if they were alive at or beyond the 3-year landmark. The patient selection process is summarized in **Supplementary Figure 1**.

### Ethics approval and reporting guideline

The study was approved by the Institutional Review Boards of Guangxi Medical University Cancer Hospital (No. KY2025841 and No. KY-2022-310), Wuzhou Red Cross Hospital (No. LL2025-112-01), and Xiangtan Central Hospital (No. KYJ-2022-002). The requirement for written informed consent was waived by all participating institutions because of the retrospective design and the use of deidentified data. The study was conducted in accordance with the 2024 revision of the Declaration of Helsinki and was reported in accordance with the TRIPOD+AI statement (15).

### Predictor collection and data preprocessing

Candidate predictors for model development were grouped into four domains: demographic characteristics; histopathologic variables; serological variables; and treatment-related variables. A complete operational dictionary listing each model-input predictor, its domain, variable type, and coding scheme is provided in **Supplementary Table 1**.

Before model development, we assessed the completeness of all candidate predictors. Variables with more than 20% missing values were excluded as a pragmatic prespecified threshold to reduce instability caused by excessive incompleteness, and missingness handling was reported according to prediction-model guidance (15–17). Missing values among retained predictors were imputed using the k-nearest neighbor method with k = 5. In brief, each incomplete case was imputed according to the values of the five most similar patients in the observed predictor space. For all internal held-out and LOCO validation workflows, imputation parameters were learned only from the corresponding training data and then applied to the held-out validation data, thereby preventing information from the internal held-out validation set or the held-out center from entering preprocessing. The distribution of missing and imputed values is shown in **Supplementary Figure 2**.

EBV DNA was primarily analyzed according to the clinically used threshold of 5000 copies/mL for consistency with the source clinical database and prior metastatic NPC studies (18). An additional sensitivity analysis using log10(EBV DNA + 1) as a continuous variable was performed to assess whether categorization materially affected discrimination or SHAP ranking. The sensitivity analysis showed that using log10(EBV DNA + 1) as a continuous variable modestly improved model discrimination, suggesting that dichotomization may have led to partial information loss.

### Outcome definition and follow-up

Patients were followed every six months. The primary endpoint was 3-year OS, defined as survival status at 3 years after the date of smNPC diagnosis. OS was calculated from the date of diagnosis to death from any cause or the date of the most recent follow-up. For the binary classification endpoint, patients who died within 3 years were labeled as events, whereas patients who were alive for at least 3 years were labeled as non-events. Patients alive with less than 3 years of follow-up were not classified as 3-year survivors and were excluded from the primary binary analysis. The marginal event rate was reported for the overall cohort, training set, internal held-out validation set, and each LOCO fold.

### Base model development

Six machine learning algorithms were used as base learners: gradient boosting decision tree (GBDT), adaptive boosting (AdaBoost), extreme gradient boosting (XGBoost), Light Gradient Boosting Machine (LightGBM), categorical boosting (CatBoost), and random forest (RF). In the primary internal held-out validation analysis, the final hyperparameter settings were fixed and were not reoptimized using the held-out validation set. The complete predefined hyperparameter settings used in the final reproducible analysis are reported in **Supplementary Table 2**.

### Ensemble strategy construction

Three ensemble strategies were then constructed on the basis of the six base learners. Hard voting aggregated the majority class predictions from the individual classifiers. Soft voting combined predicted probabilities from the base learners. Stacking used logistic regression as the meta-learner to integrate the outputs of the base models. Logistic regression was chosen as the meta-learner because it provides a parsimonious and interpretable mapping from base-learner outputs to final predicted probabilities, while reducing the risk of overfitting in a moderate-sized clinical dataset. The final meta-learner was an L2-regularized logistic regression model. To prevent data leakage, the meta-learner was trained on out-of-fold predictions generated by five-fold cross-validation within the training set. Additional predefined hyperparameter details are provided in **Supplementary Table 2**.

### Model evaluation

**Figure 1:**
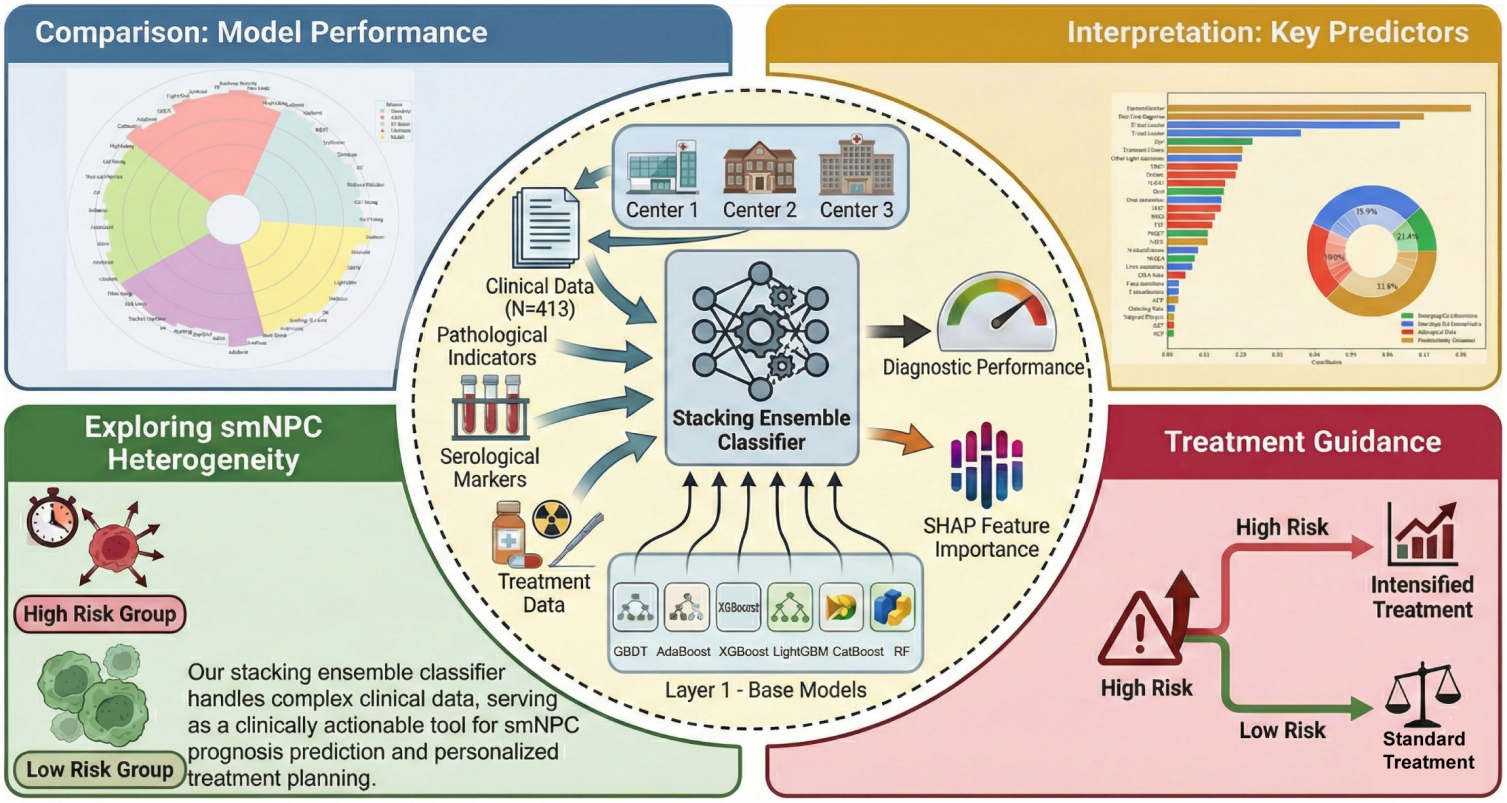
Overview of the study workflow and potential clinical application of the proposed stacking ensemble model for smNPC. Clinical, pathological, serological, and treatment-related variables from 413 patients across three centers were used to develop a stacking ensemble classifier comprising six base learners: GBDT, AdaBoost, XGBoost, LightGBM, CatBoost, and RF. The figure summarizes model development, model comparison, SHAP-based interpretation, assessment of disease heterogeneity, and risk-adapted clinical support.

The overall experimental workflow is illustrated in **Figure 1**. Model discrimination for 3-year OS was assessed primarily by receiver operating characteristic curves and the AUC. Secondary performance metrics included accuracy, precision, recall, and F1 score. Calibration was evaluated using calibration curves and the Brier score. Clinical utility was examined using decision curve analysis. Because all evaluated models were applied to the same validation cohort, paired comparisons of AUCs between the stacking model and each comparator were performed using the DeLong test or paired bootstrap resampling, with Benjamini-Hochberg false-discovery-rate adjustment for multiple comparisons. Training AUCs, validation AUCs, the optimism gap, events per variable, and bootstrap-corrected optimism were also reported to characterize overfitting risk (19–21).

To evaluate cross-center transportability, we additionally performed leave-one-center-out (LOCO) cross-validation. In each iteration, one institution served as the test cohort and the remaining two institutions were combined as the training cohort. All models were retrained in the corresponding training cohort and then tested in the held-out center. Within each LOCO iteration, missing-value imputation, feature preprocessing, model fitting, and meta-learner training were repeated independently using only the combined training centers and the same predefined hyperparameter settings. The fitted preprocessing objects and fitted models were then applied once to the held-out center. This process was repeated until each center had served once as an independent test cohort.

### Model interpretability and feature selection

To improve interpretability, we used SHAP to analyze patient-level predictor contributions of the best-performing model. For the stacking meta-learner itself, we did not rely on SHAP values for a six-input linear model. Instead, we report the directly interpretable coefficients of the L2-regularized logistic regression meta-learner, together with bootstrap-derived 95% confidence intervals, to show the relative contribution of the six base-learner outputs.

To address potential instability caused by correlated metastatic-burden predictors, pairwise associations among number of metastatic organs, number of metastatic lesions, and site-specific metastasis indicators were examined using Spearman correlation for ordered variables and phi or Cramer V statistics for categorical variables. A grouped SHAP sensitivity analysis was also performed by aggregating metastatic-burden features and site-specific metastatic features into clinically coherent predictor groups. The correlation between number of metastatic organs and number of metastatic lesions was moderate to strong, with a Spearman coefficient of 0.879. Phi coefficients between site-specific metastatic indicators ranged from 0.843 to 0.906. In the grouped SHAP analysis, metastatic-burden features remained among the most influential predictor groups, whereas site-specific metastatic features showed a lower but consistent contribution.

To identify a parsimonious predictor set, we further performed SHAP-guided iterative feature elimination with dynamic performance validation. Specifically, the least important features were sequentially removed, the model was retrained after each elimination step, and the AUC was recalculated. A tolerance threshold was used only for transparent parsimony-oriented reporting when the AUC curve reached a near-plateau; cumulative AUC values at selected predictor counts were summarized with the feature-selection results.

### Statistical analysis

Categorical variables are reported as frequencies and percentages and were compared using the chi-square test or Fisher’s exact test, as appropriate according to expected cell counts. Continuous variables were analyzed according to their distribution. Normally distributed variables are presented as mean ± standard deviation and were compared using Student’s t test, whereas nonnormally distributed variables are presented as median and interquartile range and were compared using the Mann-Whitney U test. Normality was assessed using the Shapiro-Wilk test. Baseline comparisons were intended for descriptive assessment of cohort balance; therefore, P values in **Table 1** were not adjusted for multiplicity and were interpreted as indicators of notable imbalance at the unadjusted P < 0.05 level rather than as confirmatory hypothesis tests. All statistical tests were two-sided, and P < 0.05 was considered statistically significant. Statistical analyses were performed using R version 4.2.1 and Python with the SciPy library.

**Table 1:** Comparison of clinical characteristics between the training and internal held-out validation sets.

Variable	Overall (n = 413)	Training set (n = 289)	Internal held-out validation set (n = 124)	P value
Demographic information				
Age, years	47.23 ± 11.42	47.35 ± 10.87	46.96 ± 12.64	0.767
Height, cm	165.0 (160.0–170.0)	165.0 (160.0–170.0)	165.0 (160.0–170.0)	0.434
Weight, kg	58.0 (52.0–65.0)	58.0 (52.0–65.0)	57.2 (52.9–65.0)	0.895
BMI, kg/m2	21.6 (19.7–23.6)	21.7 (19.7–23.6)	21.5 (19.7–23.6)	0.792
Sex, n (%)				0.137
Female	78 (18.9)	60 (20.8)	18 (14.5)	
Male	335 (81.1)	229 (79.2)	106 (85.5)	
Histological characteristics				
T classification, n (%)				0.818
T1-2	103 (24.9)	73 (25.3)	30 (24.2)	
T3-4	310 (75.1)	216 (74.7)	94 (75.8)	
N classification, n (%)				0.254
N0-2	174 (42.1)	127 (43.9)	47 (37.9)	
N3	239 (57.9)	162 (56.1)	77 (62.1)	
Pathology type, n (%)				0.915
WHO type I/II	39 (9.4)	27 (9.3)	12 (9.7)	
WHO type III	374 (90.6)	262 (90.7)	112 (90.3)	
Number of metastatic organs, n (%)				0.730
1	268 (64.9)	186 (64.4)	82 (66.1)	
≥2	145 (35.1)	103 (35.6)	42 (33.9)	
Number of metastatic lesions, n (%)				0.508
≤3	150 (36.3)	102 (35.3)	48 (38.7)	
>3	263 (63.7)	187 (64.7)	76 (61.3)	
Lung metastasis, n (%)				0.536
No	291 (70.5)	201 (69.6)	90 (72.6)	
Yes	122 (29.5)	88 (30.4)	34 (27.4)	
Liver metastasis, n (%)				0.483
No	283 (68.5)	195 (67.5)	88 (71.0)	
Yes	130 (31.5)	94 (32.5)	36 (29.0)	
Bone metastasis, n (%)				0.895
No	128 (31.0)	89 (30.8)	39 (31.5)	
Yes	285 (69.0)	200 (69.2)	85 (68.5)	
Other organ metastasis, n (%)				0.830
No	306 (74.1)	215 (74.4)	91 (73.4)	
Yes	107 (25.9)	74 (25.6)	33 (26.6)	
Serological data				
WBC, 10^9/L	7.1 (5.8–8.9)	7.1 (5.8–8.9)	7.0 (5.6–8.5)	0.249
NEUT, 10^9/L	4.6 (3.5–6.1)	4.6 (3.6–6.2)	4.6 (3.4–5.9)	0.305
LNC, 10^9/L	1.5 (1.2–2.0)	1.6 (1.2–2.0)	1.5 (1.1–2.0)	0.271
MONO, 10^9/L	0.6 (0.4–0.7)	0.6 (0.4–0.7)	0.6 (0.4–0.7)	0.959
HGB, g/L	131.0 (119.0–141.0)	131.0 (119.0–141.0)	131.0 (118.8–143.0)	0.634
PLT, 10^9/L	281.0 (231.0–343.0)	287.0 (237.0–349.0)	274.0 (218.8–341.2)	0.146
EBV DNA, copies/mL, n (%)				0.849
<=5000	256 (62.0)	180 (62.3)	76 (61.3)	
>5000	157 (38.0)	109 (37.7)	48 (38.7)	
ALP, U/L, n (%)				0.175
<=110	344 (83.3)	236 (81.7)	108 (87.1)	
>110	69 (16.7)	53 (18.3)	16 (12.9)	
Multimodality treatment				
MLT, n (%)				0.177
No	289 (70.0)	208 (72.0)	81 (65.3)	
Yes	124 (30.0)	81 (28.0)	43 (34.7)	
LRRT, n (%)				0.161
No	149 (36.1)	98 (33.9)	51 (41.1)	
Yes	264 (63.9)	191 (66.1)	73 (58.9)	
Immunotherapy, n (%)				0.621
No	326 (78.9)	230 (79.6)	96 (77.4)	
Yes	87 (21.1)	59 (20.4)	28 (22.6)	
Targeted therapy, n (%)				0.123
No	329 (79.7)	236 (81.7)	93 (75.0)	
Yes	84 (20.3)	53 (18.3)	31 (25.0)	
First-line regimen, n (%)				0.425
GP	87 (21.1)	60 (20.8)	27 (21.8)	
TPF	185 (44.8)	132 (45.7)	53 (42.7)	
PF	40 (9.7)	32 (11.1)	8 (6.5)	
TP	81 (19.6)	53 (18.3)	28 (22.6)	
Other	20 (4.8)	12 (4.2)	8 (6.5)	
Treatment pattern, n (%)				0.111
Systemic therapy	149 (36.1)	98 (33.9)	51 (41.1)	
MLT + primary-site radiotherapy + systemic therapy	106 (25.7)	71 (24.6)	35 (28.2)	
Primary-site radiotherapy + systemic therapy	158 (38.3)	120 (41.5)	38 (30.6)	

ALP, alkaline phosphatase; BMI, body mass index; EBV, Epstein-Barr virus; HGB, hemoglobin; LNC, lymphocyte count; LRRT, locoregional radiotherapy treatment; MLT, metastatic lesion treatment; MONO, monocyte count; NEUT, neutrophil count; PLT, platelet; WBC, white blood cell; WHO, World Health Organization. Data are presented as mean ± standard deviation, median (interquartile range), or n (%), as appropriate. Continuous variables were compared using Student’s t test or the Mann-Whitney U test according to data distribution. Categorical variables were compared using the chi-square test or Fisher’s exact test according to expected cell counts. Normality was assessed using the Shapiro-Wilk test. P values were not adjusted for multiple comparisons and were intended only for descriptive balance assessment.

## Results

### Patient characteristics

A total of 413 patients with smNPC were included in the analysis. The cohort was randomly divided into a training set of 289 patients and an internal held-out validation set of 124 patients. The numbers and proportions of 3-year death events were 248/413, 60.0%, in the overall cohort, 173/289, 59.9%, in the training set, and 75/124, 60.5%, in the internal held-out validation set. Class distributions for each center and each LOCO fold are provided in **Supplementary Table 3**. Baseline characteristics, including demographic variables, histopathologic variables, serological markers, and treatment-related factors, were compared between the two cohorts. No notable imbalances were observed at the unadjusted P < 0.05 level, supporting descriptive comparability between the training and internal held-out validation sets (**Table 1**).

### Model discrimination and overall performance

**Figure 2:**
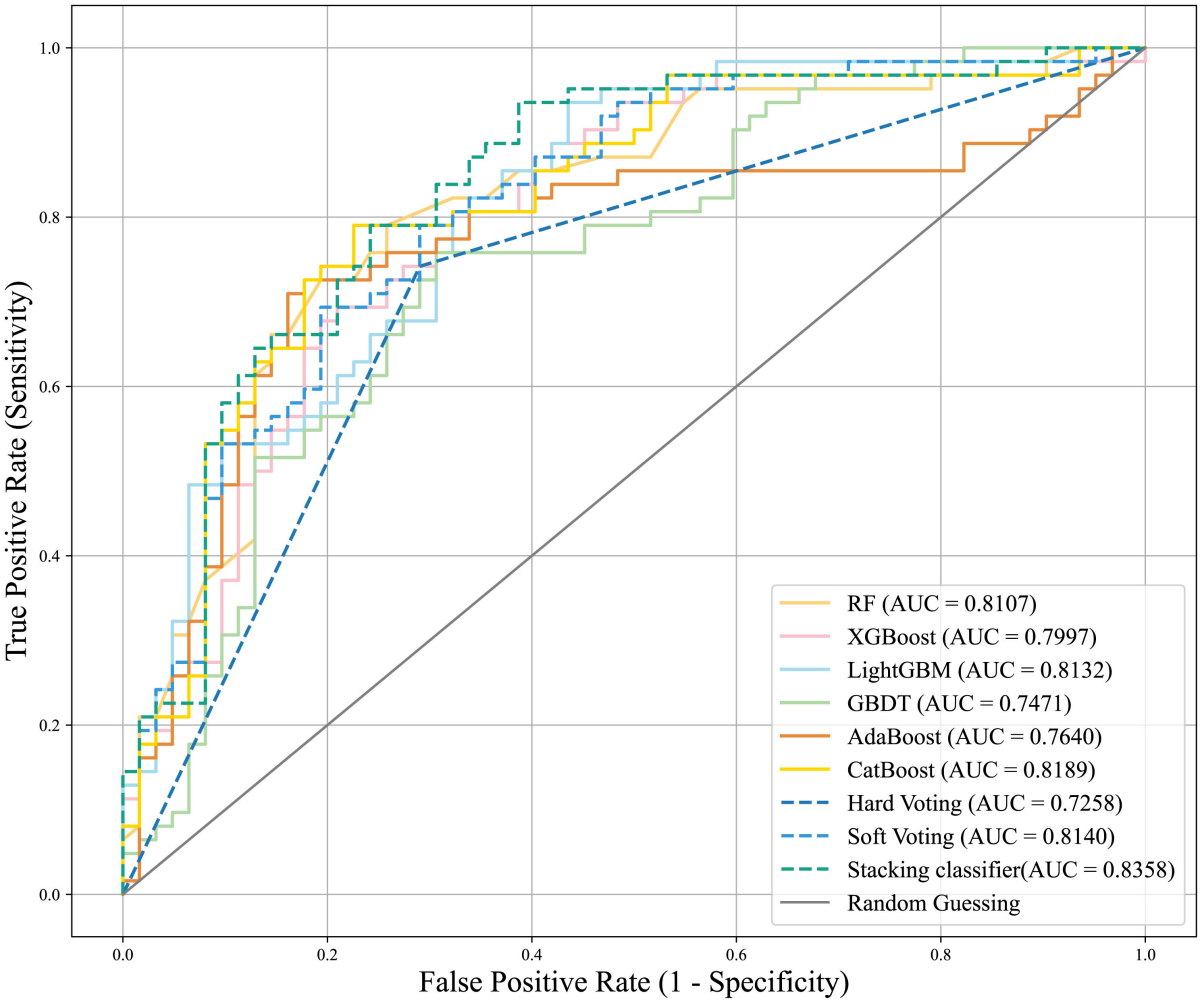
Receiver operating characteristic curves comparing the predictive performance of the evaluated machine learning models for 3-year OS.

**Figure 3:**
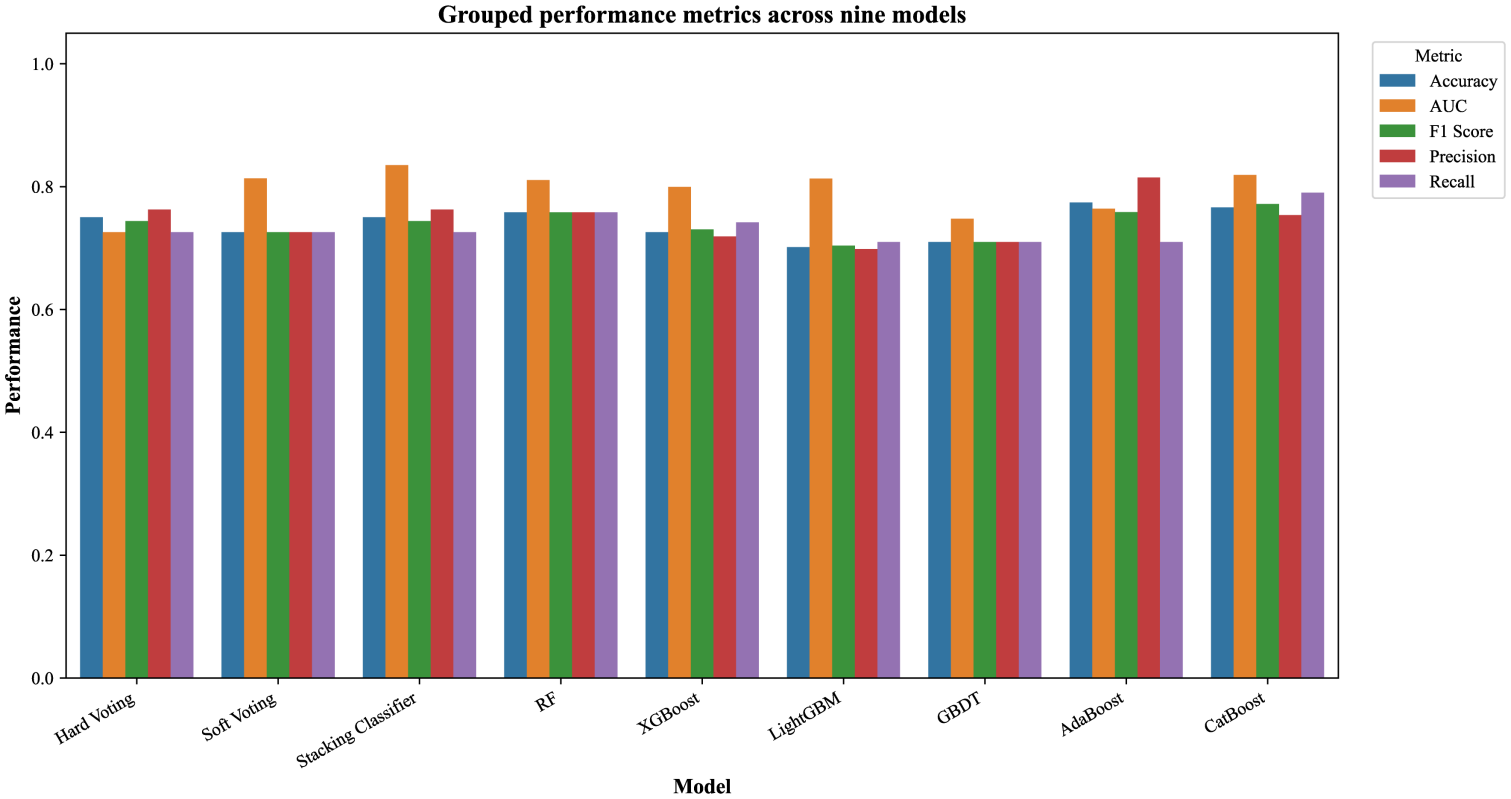
Comparative visualization of model performance metrics.

Across all evaluated models, the stacking ensemble showed the numerically highest discriminative performance for predicting 3-year OS. In the internal held-out validation set, the stacking model achieved an AUC of 0.8358, compared with 0.8107 for RF, 0.7997 for XGBoost, 0.8132 for LightGBM, 0.7471 for GBDT, 0.7640 for AdaBoost, 0.8189 for CatBoost, 0.7258 for hard voting, 0.8140 for soft voting, and 0.8150 for traditional logistic regression (**Figure 2**; **Table 2**). Paired model comparisons showed that the AUC advantage of stacking remained statistically significant after multiplicity adjustment compared with GBDT (adjusted P = 0.023), AdaBoost (adjusted P = 0.036), and hard voting (adjusted P = 0.009), whereas the differences versus RF, XGBoost, LightGBM, CatBoost, soft voting, and traditional logistic regression did not remain statistically significant. These results indicate that the gain achieved by stacking should be interpreted as a numerical improvement, with formal comparison results reported in **Supplementary Table 4**. A clearer comparative visualization of secondary classification metrics is shown in **Figure 3**; **Table 2**.

**Table 2:** Comparative predictive performance of the evaluated machine learning models.

Model	Accuracy	AUC (95% CI)	F1 score	Precision	Recall
Base models					
RF	0.7581	0.8107 (0.7680–0.8530)	0.7381	0.7581	0.7281
XGBoost	0.7258	0.7997 (0.7540–0.8450)	0.7302	0.7188	0.7419
LightGBM	0.7016	0.8132 (0.7710–0.8550)	0.7040	0.6984	0.7097
GBDT	0.7097	0.7471 (0.6980–0.7960)	0.7097	0.7097	0.7097
AdaBoost	0.7742	0.7640 (0.7170–0.8110)	0.7586	0.8148	0.7097
CatBoost	0.7661	0.8189 (0.7760–0.8620)	0.7717	0.7538	0.7903
Ensemble models					
Hard voting	0.7500	0.7258 (0.6750–0.7760)	0.7437	0.7624	0.7258
Soft voting	0.7258	0.8140 (0.7720–0.8560)	0.7252	0.7256	0.7251
Stacking classifier	0.7500	0.8358 (0.7950–0.8760)	0.7438	0.7627	0.7438
Traditional logistic regression	0.7500	0.8150 (0.7730–0.8570)	0.7520	0.7460	0.7581

AdaBoost, adaptive boosting; AUC, area under the curve; CatBoost, categorical boosting; CI, confidence interval; GBDT, gradient boosting decision tree; LightGBM, Light Gradient Boosting Machine; RF, random forest; XGBoost, extreme gradient boosting. All metrics are reported to four decimal places for consistency.

Overfitting diagnostics showed a training AUC of 0.9150 and a validation AUC of 0.8358 for the final stacking classifier, yielding an optimism gap of 0.0792. With 173 3-year death events in the training set and 27 candidate predictors, the events-per-variable estimate was 6.4. Bootstrap validation yielded an apparent AUC of 0.9165, a mean optimism of 0.0750, and an optimism-corrected AUC of 0.8415 (**Supplementary Table 5**).

### Cross-center transportability, calibration, and clinical utility

Consistent findings were observed in LOCO validation. The stacking model maintained comparable values for AUC, accuracy, recall, and F1 score across all three held-out centers, supporting its transportability across institutions within this multicenter cohort (**Supplementary Table 6**). All preprocessing, imputation, model fitting, and meta-learner training steps were repeated within each training fold using the same predefined hyperparameter settings, and no information from the held-out center was used during training.

Calibration analysis showed that the stacking model achieved the best agreement between predicted and observed 3-year OS probabilities among the evaluated models. Its calibration curve lay closest to the ideal reference line, and it produced the lowest Brier score of 0.1563 (**Supplementary Figure 3A**). Decision curve analysis further showed that the stacking model yielded the highest net benefit across clinically relevant threshold probabilities of 0.2 to 0.6, compared with the alternative machine learning models as well as the treat-all and treat-none strategies (**Supplementary Figure 3B**).

### Model interpretability and feature importance

**Figure 4:**
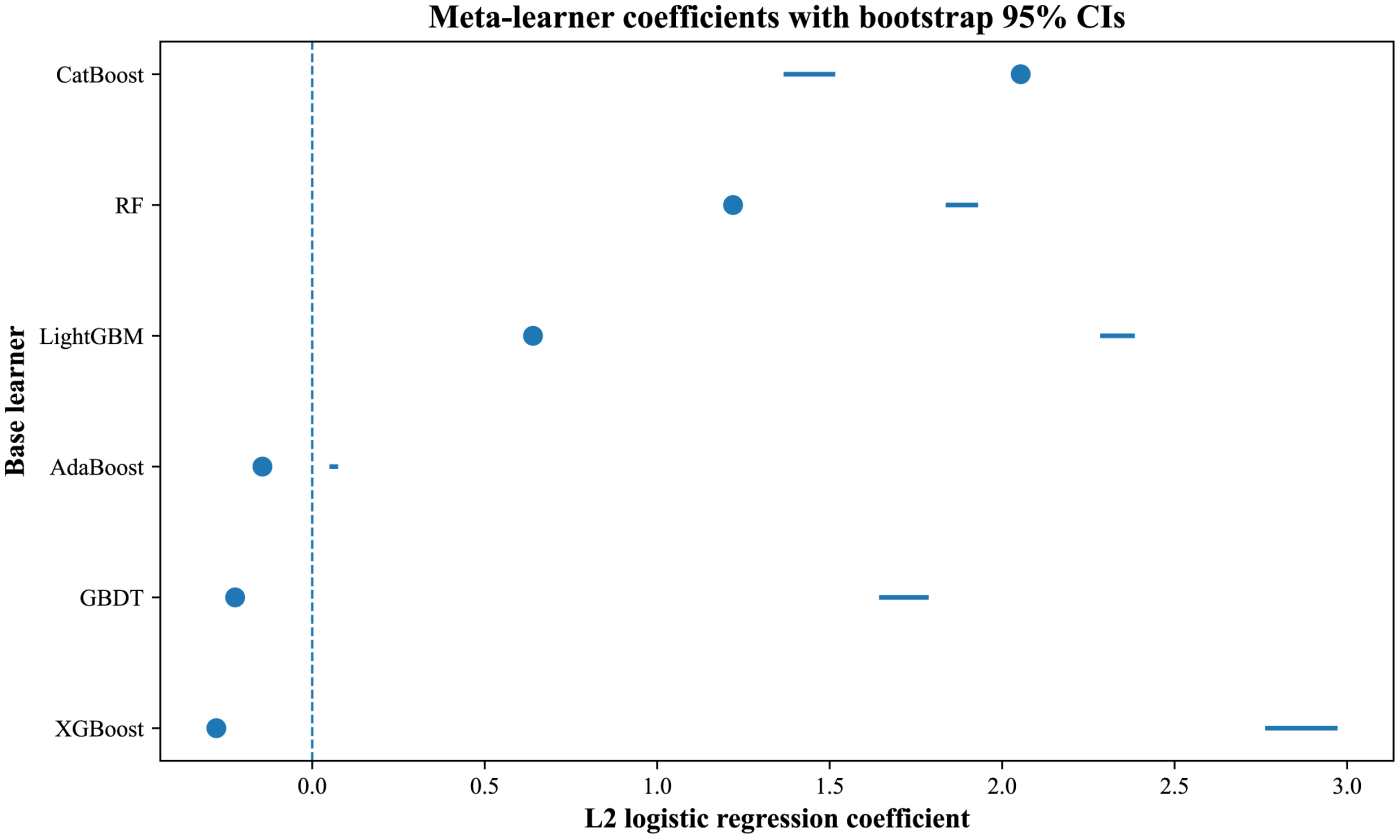
Coefficient plot of the L2-regularized logistic regression meta-learner. Coefficients and bootstrap-derived 95% confidence intervals show the relative contribution of the six base-learner probability outputs to the final stacking prediction.

To clarify the internal decision structure of the stacking ensemble, we reported the coefficients of the L2-regularized logistic regression meta-learner rather than SHAP values for the six base-learner outputs. This more directly reflects how the meta-learner weighted the probability outputs of the base models. The coefficient plot, with bootstrap-derived 95% confidence intervals, is shown in **Figure 4**.

**Figure 5:**
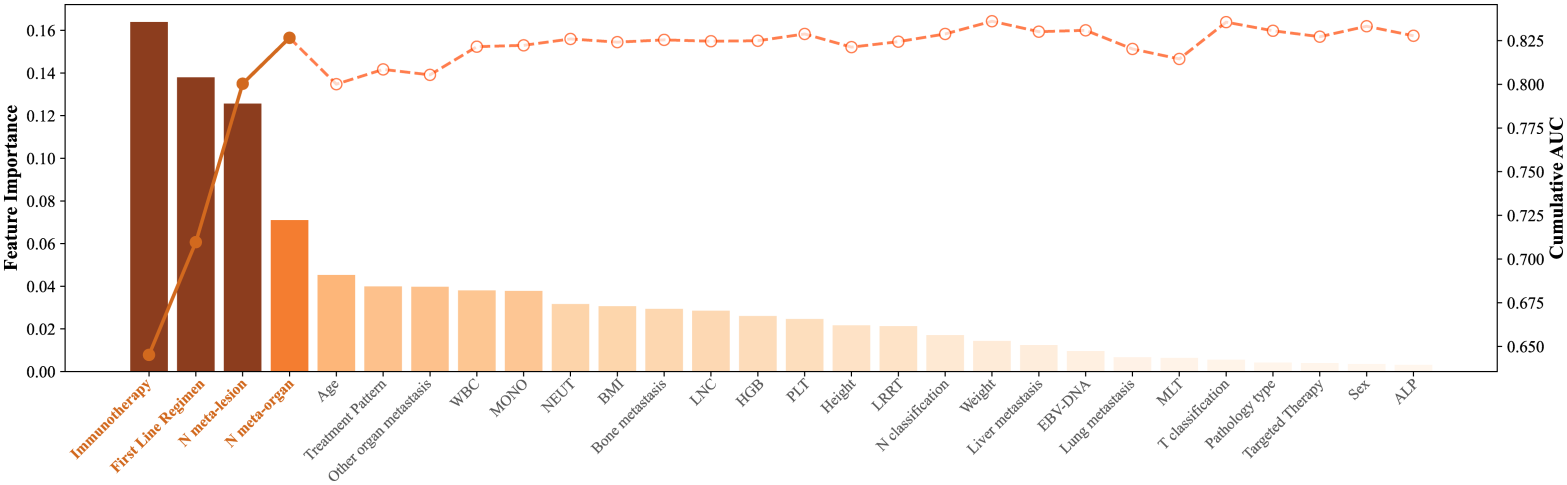
SHAP-guided cumulative feature-addition analysis. The symbol k denotes the number of predictors retained in the cumulative feature-addition analysis. Cumulative AUCs were 0.828 at k = 4, 0.827 at k = 10, 0.825 at k = 15, and 0.826 at k = 27, indicating a near-flat performance curve after approximately four predictors.

SHAP-guided iterative feature elimination showed that model performance was relatively stable across a broad range of predictor counts. The cumulative AUCs at selected predictor counts were 0.828 at k = 4, ending with N meta-organ; 0.827 at k = 10, ending with BMI; 0.825 at k = 15, ending with Height; and 0.826 at k = 27, ending with ALP. Therefore, k = 4 was described as a parsimonious choice within a near-flat performance region (**Figure 5**; **Supplementary Table 7**).

**Figure 6:**
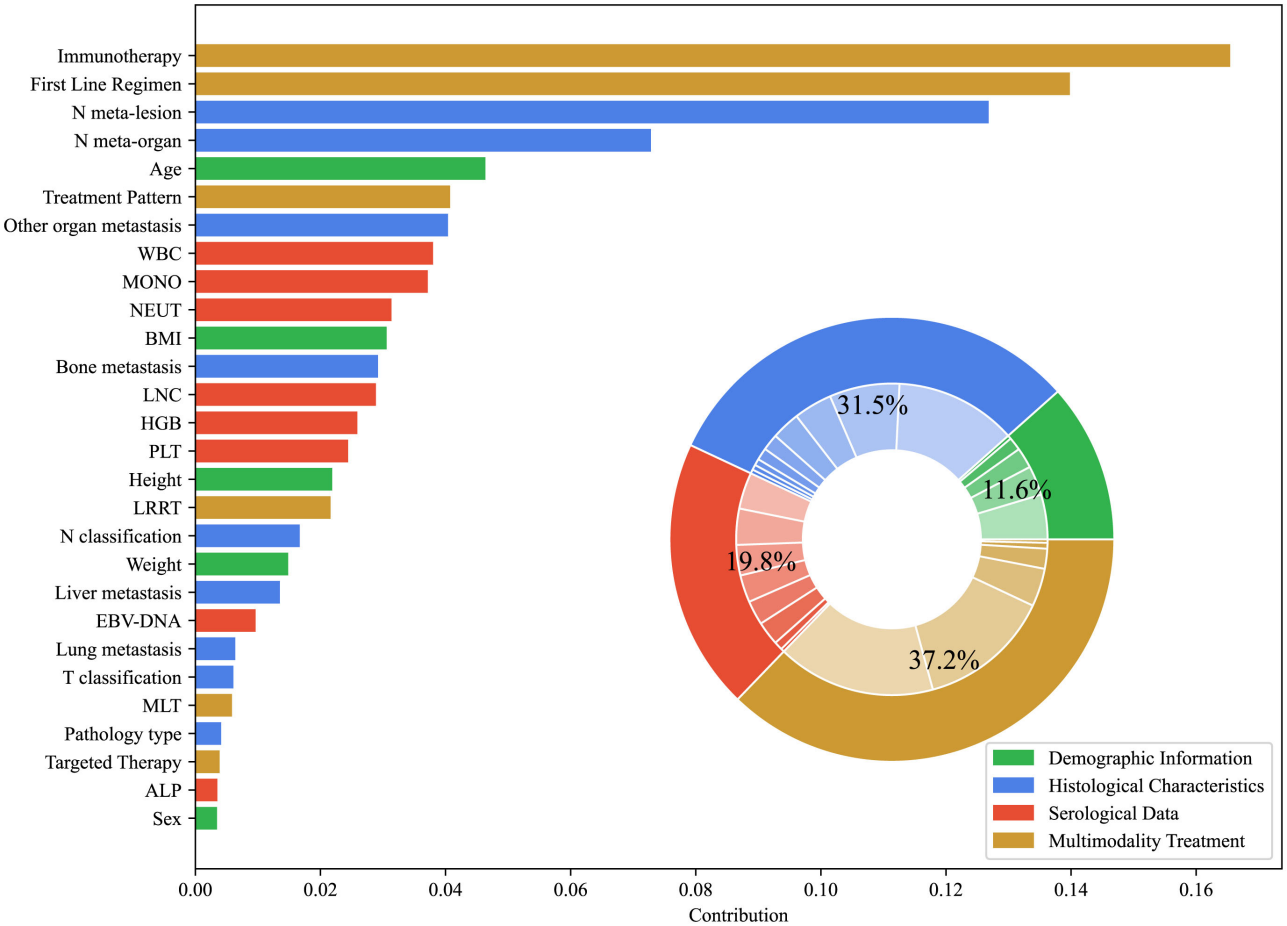
Integrated plot showing SHAP-based feature importance in the stacking model. Treatment-related variables contributed the largest proportion of overall model importance at 37.2%, followed by histopathologic variables at 31.5%, serological variables at 19.8%, and demographic characteristics at 11.6%. The four most influential individual predictors were immunotherapy, first-line regimen, number of metastatic lesions, and number of metastatic organs.

Global SHAP analysis showed that treatment-related variables contributed the largest share of overall model importance at 37.2%, followed by histopathologic variables at 31.5%, serological variables at 19.8%, and demographic characteristics at 11.6% (**Figure 6**). At the individual feature level, immunotherapy, first-line regimen, number of metastatic lesions, and number of metastatic organs showed the highest SHAP values, indicating that they were influential determinants of individualized 3-year OS prediction.

## Discussion

In this multicenter retrospective study, we developed and validated a stacking ensemble machine learning model for predicting 3-year OS in patients with smNPC. The stacking model achieved the numerically highest internal held-out validation AUC among the evaluated approaches, showed favorable calibration, yielded high net benefit in decision curve analysis, and maintained stable performance in LOCO validation. After formal paired comparisons, the magnitude and statistical significance of differences between stacking and alternative models should be interpreted according to the adjusted P values rather than by AUC ranking alone. SHAP-based interpretation further showed that immunotherapy, first-line regimen, number of metastatic lesions, and number of metastatic organs were influential predictors.

The performance of stacking is biologically and methodologically plausible. Patients with smNPC represent a highly heterogeneous population in whom prognosis depends on multiple domains of information, including tumor burden, metastatic distribution, systemic condition, and treatment selection (6). Under these conditions, reliance on a single statistical framework may lead to underfitting of complex nonlinear associations or oversensitivity to the idiosyncrasies of a specific algorithm. By contrast, stacking learns how to combine the complementary strengths of several base learners through a meta-learner, which can improve robustness and discrimination in clinically heterogeneous datasets (13, 14). The stable performance observed across the center-based validation analyses further supports the potential value of this approach in real-world multicenter settings.

Our findings are consistent with the growing literature supporting stacking-based prediction models in oncology. Ling et al. used a stacking classifier to integrate computed tomography radiomics and clinical biomarkers for predicting lymph node metastasis in locally advanced gastric cancer after neoadjuvant chemotherapy and reported favorable performance (22). Li et al. also showed that an ensemble classifier integrating radiomics with clinical radiological variables improved preoperative prediction of pathological invasiveness in lung adenocarcinoma presenting as part-solid nodules (23). Together with the present study, these data suggest that stacking is particularly well suited to multimodal oncologic datasets that contain nonlinear relationships and interaction effects that are difficult to model with a single method.

SHAP analysis highlighted immunotherapy as an influential predictor, which is consistent with the evolving treatment landscape of recurrent or metastatic nasopharyngeal carcinoma. However, because treatment pattern and immunotherapy were not randomly assigned, their model importance should not be interpreted as evidence of treatment efficacy. These variables may capture treatment exposure, clinician judgment, calendar period, drug availability, baseline performance status, disease burden, and confounding by indication. We therefore interpret treatment-related variables as predictive markers within the model rather than as causal treatment-effect estimates.

The importance of first-line regimen, number of metastatic lesions, and number of metastatic organs is also clinically interpretable. Chemotherapy remains a cornerstone of treatment for smNPC, and prior studies have shown meaningful survival differences between commonly used platinum-based regimens (4, 24, 25). Metastatic burden is another established determinant of outcome. Since the original description of oligometastasis by Hellman and Weichselbaum (26), increasing attention has been paid to the prognostic relevance of both lesion count and organ count. Recent staging refinements and retrospective studies suggest that a limited metastatic burden identifies a subgroup with more favorable biology and a greater likelihood of benefiting from aggressive local treatment (27–30). Our model supports this concept by showing that both the number of metastatic lesions and the number of involved organs carry substantial prognostic information.

From a clinical perspective, the proposed model may help support individualized prognostic assessment in patients with smNPC (31, 32). In practice, such a tool could assist multidisciplinary teams in identifying patients at relatively high risk who may benefit from closer multidisciplinary review and individualized follow-up planning (33, 34). At the same time, it may help contextualize prognosis in lower-risk patients. However, the model is intended to complement clinical judgment rather than replace it (35, 36).

Several limitations should be acknowledged. First, the retrospective design introduces the possibility of selection bias, information bias, residual confounding, and treatment-confounding by indication. In particular, treatment pattern and immunotherapy may reflect baseline prognosis and clinician judgment rather than causal treatment effects. Second, the endpoint was overall survival, and causes of death were not consistently available. Therefore, competing risks from non-cancer deaths were not explicitly modeled. Given the relatively young median age of approximately 47 years, this limitation may have had a modest influence, but future studies with cause-specific mortality data are warranted. Third, although the multicenter design and leave-one-center-out validation supported transportability across participating institutions, prospective external validation remains necessary before clinical implementation.

## Conclusion

In conclusion, this multicenter retrospective study showed that a stacking ensemble machine learning model may provide an interpretable approach for predicting 3-year OS in smNPC. The model should be regarded as a risk-stratification tool with potential clinical utility, rather than as evidence for causal treatment effects, and should be further evaluated in prospective external cohorts before routine clinical use.

## Data Availability

The datasets generated and/or analyzed during the current study are not publicly available because they contain de-identified but potentially sensitive patient-level clinical data and are subject to institutional and ethical restrictions. De-identified data supporting the findings of this study may be made available from the corresponding authors upon reasonable request and after approval by the participating institutions/IRBs. Requests should be directed to Qiwen Duan (duanqiwen603@gmail.com) and Guanxun Cheng (chengguanxun@outlook.com).

## Supplementary material

Supplementary Figure 1 Flowchart of patient selection and cohort construction. A total of 616 patients with smNPC from three centers were screened for eligibility between January 2010 and January 2023.

Supplementary Figure 2 Pareto chart showing the distribution of missing values and the status of imputation among retained predictors.

Supplementary Figure 3 **(A)** Calibration curves for 3-year OS prediction across the evaluated models. The stacking model, with a Brier score of 0.1563, showed the closest agreement with ideal calibration. **(B)** Decision curve analysis for 3-year OS prediction across the evaluated models. The stacking model yielded the highest net benefit at clinically relevant threshold probabilities of 0.2 to 0.6.
