# Supplementary figures and images for: Predicting overall survival in synchronous metastatic nasopharyngeal carcinoma using a stacking ensemble machine learning model: a multicenter retrospective study
Source: Front Oncol. 2026 Jul 1;16:1866690. doi: 10.3389/fonc.2026.1866690 (PMC13368551; doi:10.3389/fonc.2026.1866690)

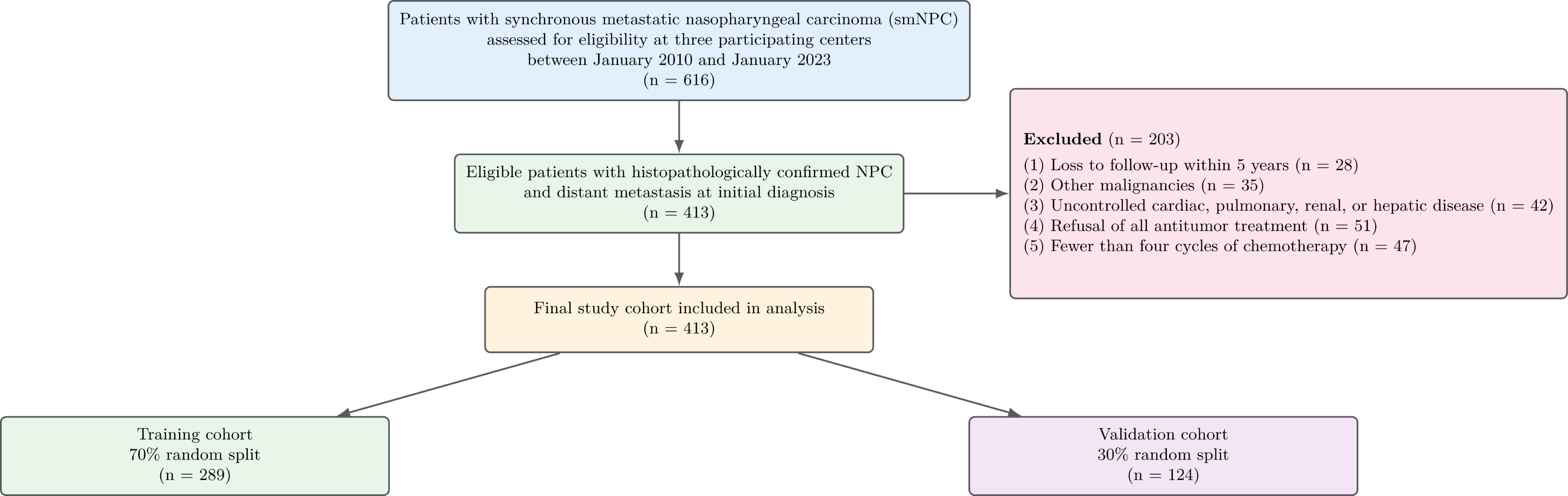

Supplement: Supplementary Figure 1 — Flowchart of patient selection and cohort construction. A total of 616 patients with smNPC from three centers were screened for eligibility between January 2010 and January 2023. [file Image1.tif]

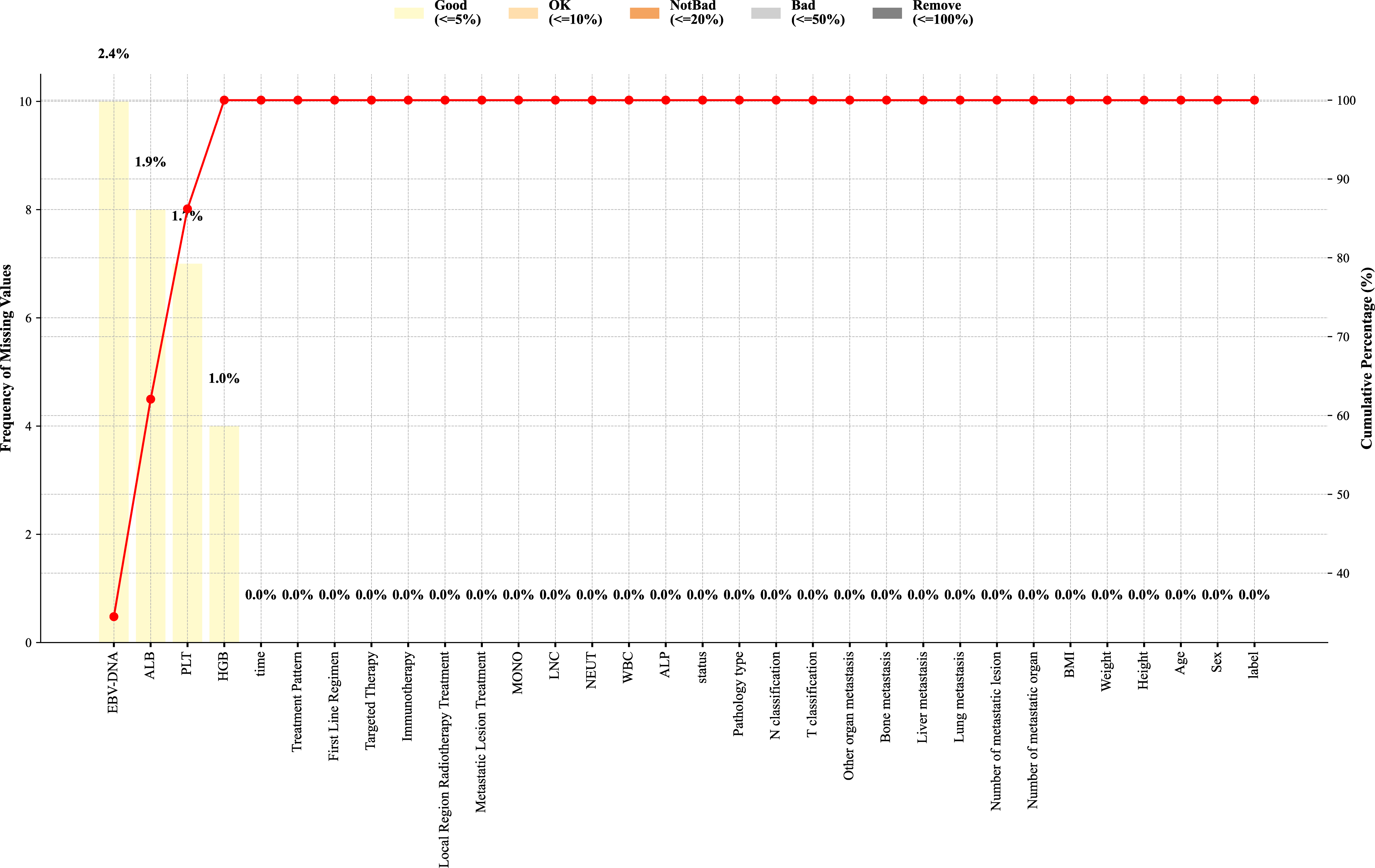

Supplement: Supplementary Figure 2 — Pareto chart showing the distribution of missing values and the status of imputation among retained predictors. [file Image2.tif]

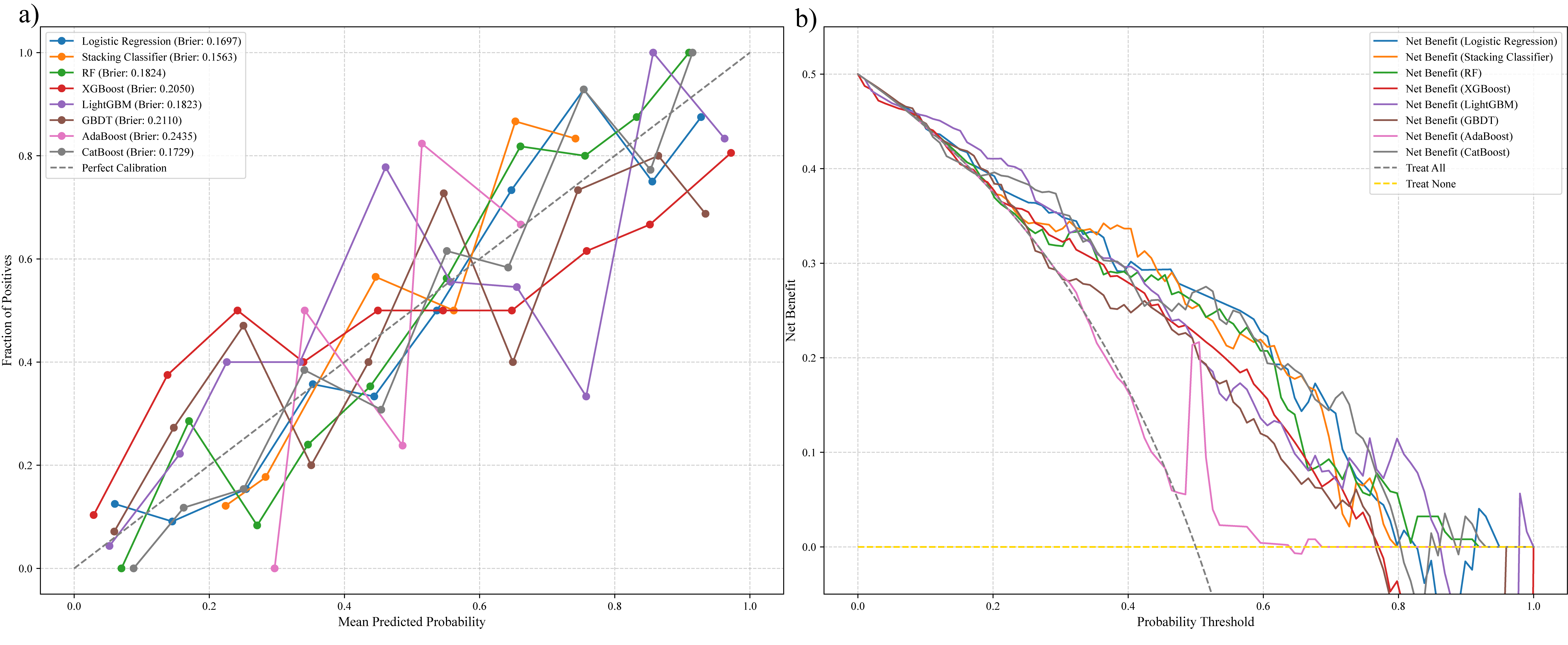

Supplement: Supplementary Figure 3 — (A) Calibration curves for 3-year OS prediction across the evaluated models. The stacking model, with a Brier score of 0.1563, showed the closest agreement with ideal calibration. (B) Decision curve analysis for 3-year OS prediction across the evaluated models. The stacking model yielded the highest net benefit at clinically relevant threshold probabilities of 0.2 to 0.6. [file Image3.tif]
