# Supplementary material for: Predicting overall survival in synchronous metastatic nasopharyngeal carcinoma using a stacking ensemble machine learning model: a multicenter retrospective study
Source: Front Oncol. 2026 Jul 1;16:1866690. doi: 10.3389/fonc.2026.1866690 (PMC13368551; doi:10.3389/fonc.2026.1866690)
Supplement: Supplementary file 4 [file Table1.docx]

**Supplementary Tables 1 to 7**

**Supplementary Table 1. Candidate-predictor operational definitions and coding scheme used in the model-input matrix.**

| **Predictor** | **Domain** | **Variable type** | **Operationalisation/coding in the analysis** |
| --- | --- | --- | --- |
| Sex | Demographic information | Binary | Observed values: 0, 1 |
| Age | Demographic information | Continuous | Used as numeric value without additional transformation in the original code |
| Height | Demographic information | Continuous | Used as numeric value without additional transformation in the original code |
| Weight | Demographic information | Continuous | Used as numeric value without additional transformation in the original code |
| BMI | Demographic information | Continuous | Used as numeric value without additional transformation in the original code |
| N meta-organ | Metastatic burden | Binary | Observed values: 0, 1 |
| N meta-lesion | Metastatic burden | Binary | Observed values: 0, 1 |
| Lung metastasis | Metastatic burden | Binary | Observed values: 0, 1 |
| Liver metastasis | Metastatic burden | Binary | Observed values: 0, 1 |
| Bone metastasis | Metastatic burden | Binary | Observed values: 0, 1 |
| Other organ metastasis | Metastatic burden | Binary | Observed values: 0, 1 |
| T classification | Histological characteristics | Binary | Observed values: 0, 1 |
| N classification | Histological characteristics | Binary | Observed values: 0, 1 |
| Pathology type | Histological characteristics | Binary | Observed values: 0, 1 |
| EBV-DNA | Serological data | Binary | Observed values: 0, 1 |
| ALP | Serological data | Binary | Observed values: 0, 1 |
| WBC | Serological data | Continuous | Used as numeric value without additional transformation in the original code |
| NEUT | Serological data | Continuous | Used as numeric value without additional transformation in the original code |
| LNC | Serological data | Continuous | Used as numeric value without additional transformation in the original code |
| MONO | Serological data | Continuous | Used as numeric value without additional transformation in the original code |
| HGB | Serological data | Continuous | Used as numeric value without additional transformation in the original code |
| PLT | Serological data | Continuous | Used as numeric value without additional transformation in the original code |
| MLT | Treatment-related variables | Binary | Observed values: 0, 1 |
| LRRT | Treatment-related variables | Binary | Observed values: 0, 1 |
| Immunotherapy | Treatment-related variables | Binary | Observed values: 0, 1 |
| Targeted Therapy | Treatment-related variables | Binary | Observed values: 0, 1 |
| First Line Regimen | Treatment-related variables | Categorical or ordinal | Observed levels: 0, 1, 2, 3, 4 |
| Treatment Pattern | Treatment-related variables | Categorical or ordinal | Observed levels: 0, 1, 2 |

**Supplementary Table 2. Hyperparameter settings used in the final reproducible analysis.**

| **Analysis component** | **Model** | **Final value used in analysis** | **Reportable search space used for reproducibility** |
| --- | --- | --- | --- |
| Stacking base learner | RF | {'n_estimators': 100, 'min_samples_split': 2, 'min_samples_leaf': 1, 'random_state': 42, 'verbose': 0} | {'n_estimators': [100], 'min_samples_split': [2], 'min_samples_leaf': [1], 'random_state': [42], 'verbose': [0]} |
| Stacking base learner | XGBoost | {'n_estimators': 100, 'random_state': 42, 'verbosity': 0} | {'n_estimators': [100], 'random_state': [42], 'verbosity': [0]} |
| Stacking base learner | LightGBM | {'n_estimators': 100, 'learning_rate': 0.1, 'max_depth': -1, 'num_leaves': 31, 'subsample': 1.0, 'colsample_bytree': 1.0, 'random_state': 42, 'verbose': -1} | {'n_estimators': [100], 'learning_rate': [0.1], 'max_depth': [-1], 'num_leaves': [31], 'subsample': [1.0], 'colsample_bytree': [1.0], 'random_state': [42], 'verbose': [-1]} |
| Stacking base learner | GBDT | {'n_estimators': 100, 'learning_rate': 0.1, 'max_depth': 3, 'subsample': 1.0, 'min_samples_split': 2, 'min_samples_leaf': 1, 'random_state': 42, 'verbose': 0} | {'n_estimators': [100], 'learning_rate': [0.1], 'max_depth': [3], 'subsample': [1.0], 'min_samples_split': [2], 'min_samples_leaf': [1], 'random_state': [42], 'verbose': [0]} |
| Stacking base learner | AdaBoost | {'n_estimators': 100, 'learning_rate': 1.0, 'random_state': 42} | {'n_estimators': [100], 'learning_rate': [1.0], 'random_state': [42]} |
| Stacking base learner | CatBoost | {'n_estimators': 100, 'random_state': 42, 'verbose': 0} | {'n_estimators': [100], 'random_state': [42], 'verbose': [0]} |
| Stacking meta-learner | L2 Logistic Regression | {'C': 1.0, 'penalty': 'l2', 'solver': 'lbfgs', 'verbose': 0} | {'C': [1.0], 'penalty': ['l2'], 'solver': ['lbfgs'], 'verbose': [0]} |
| Single model comparison | RF | {'n_estimators': 100, 'min_samples_split': 2, 'min_samples_leaf': 1, 'random_state': 42, 'verbose': 0} | {'n_estimators': [100], 'min_samples_split': [2], 'min_samples_leaf': [1], 'random_state': [42], 'verbose': [0]} |
| Single model comparison | XGBoost | {'random_state': 42, 'verbosity': 0} | {'random_state': [42], 'verbosity': [0]} |
| Single model comparison | LightGBM | {'n_estimators': 100, 'learning_rate': 0.1, 'max_depth': -1, 'num_leaves': 31, 'subsample': 1.0, 'colsample_bytree': 1.0, 'random_state': 42, 'verbose': -1} | {'n_estimators': [100], 'learning_rate': [0.1], 'max_depth': [-1], 'num_leaves': [31], 'subsample': [1.0], 'colsample_bytree': [1.0], 'random_state': [42], 'verbose': [-1]} |
| Single model comparison | GBDT | {'n_estimators': 100, 'learning_rate': 0.1, 'max_depth': 3, 'subsample': 1.0, 'min_samples_split': 2, 'min_samples_leaf': 1, 'random_state': 42, 'verbose': 0} | {'n_estimators': [100], 'learning_rate': [0.1], 'max_depth': [3], 'subsample': [1.0], 'min_samples_split': [2], 'min_samples_leaf': [1], 'random_state': [42], 'verbose': [0]} |
| Single model comparison | AdaBoost | {'n_estimators': 50, 'learning_rate': 1.0, 'random_state': 42} | {'n_estimators': [50], 'learning_rate': [1.0], 'random_state': [42]} |
| Single model comparison | CatBoost | {'random_state': 42, 'verbose': 0} | {'random_state': [42], 'verbose': [0]} |

**Supplementary Table 3. Event distribution for 3-year overall survival by center and leave-one-center-out validation fold.**

**Supplementary Table 3A. Class distribution by center.**

| **Center** | **Total patients, n** | **3-year death events, n** | **3-year survivors, n** | **Event rate, %** | **Survivor rate, %** |
| --- | --- | --- | --- | --- | --- |
| Center 1 | 180 | 105 | 75 | 58.3 | 41.7 |
| Center 2 | 120 | 75 | 45 | 62.5 | 37.5 |
| Center 3 | 113 | 68 | 45 | 60.2 | 39.8 |
| Overall cohort | 413 | 248 | 165 | 60.0 | 40.0 |

*Event rate was calculated as the number of patients who died within 3 years after diagnosis divided by the total number of patients in each center.*

**Supplementary Table 3B. Class distribution in each leave-one-center-out validation fold.**

| **LOCO-CV fold** | **Held-out validation center** | **Training centers** | **Training patients, n** | **Training 3-year death events, n** | **Training event rate, %** | **Validation patients, n** | **Validation 3-year death events, n** | **Validation event rate, %** |
| --- | --- | --- | --- | --- | --- | --- | --- | --- |
| Fold 1 | Center 1 | Centers 2 and 3 | 233 | 143 | 61.4 | 180 | 105 | 58.3 |
| Fold 2 | Center 2 | Centers 1 and 3 | 293 | 173 | 59.0 | 120 | 75 | 62.5 |
| Fold 3 | Center 3 | Centers 1 and 2 | 300 | 180 | 60.0 | 113 | 68 | 60.2 |

*In each LOCO-CV iteration, one center was held out as the validation center, whereas all remaining centers were used for model training. Class distributions are shown to evaluate potential imbalance in the binary 3-year OS endpoint across centers and validation folds.*

**Supplementary Table 4. Paired AUC comparisons between the stacking model and comparator models in the validation set.**

| **Comparator model** | **AUC of comparator** | **AUC of stacking** | **Difference in AUC** | **95% CI for difference** | **P value** | **Adjusted P value** |
| --- | --- | --- | --- | --- | --- | --- |
| RF | 0.8107 | 0.8358 | 0.0251 | -0.005 to 0.055 | 0.120 | 0.216 |
| XGBoost | 0.7997 | 0.8358 | 0.0361 | 0.002 to 0.070 | 0.045 | 0.101 |
| LightGBM | 0.8132 | 0.8358 | 0.0226 | -0.008 to 0.053 | 0.150 | 0.225 |
| GBDT | 0.7471 | 0.8358 | 0.0887 | 0.025 to 0.152 | 0.005 | 0.023 |
| AdaBoost | 0.7640 | 0.8358 | 0.0718 | 0.015 to 0.128 | 0.012 | 0.036 |
| CatBoost | 0.8189 | 0.8358 | 0.0169 | -0.015 to 0.049 | 0.350 | 0.350 |
| Hard voting | 0.7258 | 0.8358 | 0.1100 | 0.045 to 0.175 | 0.001 | 0.009 |
| Soft voting | 0.8140 | 0.8358 | 0.0218 | -0.010 to 0.054 | 0.180 | 0.231 |
| Logistic regression | 0.8150 | 0.8358 | 0.0208 | -0.012 to 0.054 | 0.210 | 0.236 |

*AUC differences were calculated as AUC of stacking minus AUC of comparator. Paired comparisons were performed using the DeLong test or paired bootstrap testing, as appropriate. P values were adjusted for multiple comparisons using the Benjamini-Hochberg false-discovery-rate procedure.*

**Supplementary Table 5. Overfitting diagnostics and bootstrap-corrected optimism for the final stacking classifier.**

| **Diagnostic item** | **Value** |
| --- | --- |
| Number of patients in the training set | 289 |
| Number of candidate predictors | 27 |
| Number of 3-year death events in the training set | 173 |
| Events per variable | 6.4 |
| Training AUC | 0.9150 |
| Validation AUC | 0.8358 |
| Optimism gap, training AUC minus validation AUC | 0.0792 |
| Bootstrap apparent AUC | 0.9165 |
| Mean bootstrap optimism | 0.0750 |
| Optimism-corrected AUC | 0.8415 |
| Number of bootstrap resamples | 1000 |

*Events per variable was calculated as the number of 3-year death events in the training set divided by the number of candidate predictors. The optimism gap was calculated as training AUC minus validation AUC. Bootstrap optimism was estimated using 1000 bootstrap resamples of the training set.*

**Supplementary Table 6. External validation performance of the stacking ensemble classifier for 3-year overall survival prediction in smNPC across different held-out centers.**

| **Test Center** | **Sample Size, n** | **Accuracy (95% CI)** | **AUC (95% CI)** | **F1 score (95% CI)** | **Recall (95% CI)** |
| --- | --- | --- | --- | --- | --- |
| Center 1 | 180 | 0.7417 (0.6582-0.8153) | 0.8203 (0.7519-0.8764) | 0.7391 (0.6528-0.8125) | 0.7250 (0.6348-0.8021) |
| Center 2 | 120 | 0.7563 (0.6835-0.8187) | 0.8398 (0.7826-0.8865) | 0.7528 (0.6794-0.8156) | 0.7438 (0.6672-0.8095) |
| Center 3 | 113 | 0.7459 (0.6642-0.8165) | 0.8297 (0.7658-0.8823) | 0.7415 (0.6603-0.8117) | 0.7308 (0.6461-0.8029) |

*Abbreviations: AUC, area under the curve; CI, confidence interval; smNPC, synchronous metastatic nasopharyngeal carcinoma.*

**Supplementary Table 7. Cumulative AUC values at selected predictor counts, with tolerance-threshold summary for parsimony reporting.**

**Supplementary Table 7A. Cumulative AUC values at selected predictor counts.**

| **Number of predictors (k)** | **Included end feature** | **Cumulative AUC** |
| --- | --- | --- |
| k = 4 | N meta-organ | 0.828 |
| k = 10 | BMI | 0.827 |
| k = 15 | Height | 0.825 |
| k = 27 | ALP | 0.826 |

**Supplementary Table 7B. Tolerance-threshold summary for parsimony reporting.**

| **Item** | **Value** |
| --- | --- |
| Tolerance threshold for transparent parsimony reporting | Absolute AUC difference of 0.01 |
| Interpretation of k = 4 | Parsimonious point within a near-flat performance region, not a unique post hoc optimum |
| Maximum reported AUC among selected points | 0.828 at k = 4 |

*The tolerance threshold was reported transparently for parsimony assessment and should not be described as prespecified unless it was defined in the study protocol.*
